# A novel inhibitor of the PI3K/Akt pathway based on the structure of inositol 1,3,4,5,6-pentakisphosphate

**DOI:** 10.1038/sj.bjc.6605408

**Published:** 2010-01-05

**Authors:** M Falasca, D Chiozzotto, H Y Godage, M Mazzoletti, A M Riley, S Previdi, B V L Potter, M Broggini, T Maffucci

**Affiliations:** 1 Queen Mary University of London, Barts and The London School of Medicine and Dentistry, Blizard Institute of Cell and Molecular Science, Centre for Diabetes, Inositide Signalling Group, 4 Newark Street, London E1 2AT, UK; 2 Wolfson Laboratory of Medicinal Chemistry, Department of Pharmacy and Pharmacology, University of Bath, Claverton Down, Bath BA2 7AY, UK; 3 Laboratory of Molecular Pharmacology, Department of Oncology, Istituto di Ricerche Farmacologiche Mario Negri, Via La Masa 19, Milan 20156, Italy

**Keywords:** phosphoinositide 3-kinase, inositol polyphosphates, protein kinase B-Akt, 3-phosphoinositide-dependent protein kinase 1, apoptosis

## Abstract

**Background:**

Owing to its role in cancer, the phosphoinositide 3-kinase (PI3K)/Akt pathway is an attractive target for therapeutic intervention. We previously reported that the inhibition of Akt by inositol 1,3,4,5,6-pentakisphosphate (InsP_5_) results in anti-tumour properties. To further develop this compound we modified its structure to obtain more potent inhibitors of the PI3K/Akt pathway.

**Methods:**

Cell proliferation/survival was determined by cell counting, sulphorhodamine or acridine orange/ethidium bromide assay; Akt activation was determined by western blot analysis. *In vivo* effect of compounds was tested on PC3 xenografts, whereas *in vitro* activity on kinases was determined by SelectScreen Kinase Profiling Service.

**Results:**

The derivative 2-*O*-benzyl-*myo*-inositol 1,3,4,5,6-pentakisphosphate (2-*O*-Bn-InsP_5_) is active towards cancer types resistant to InsP_5_ *in vitro* and *in vivo*. 2-*O*-Bn-InsP_5_ possesses higher pro-apoptotic activity than InsP_5_ in sensitive cells and enhances the effect of anti-cancer compounds. 2-*O*-Bn-InsP_5_ specifically inhibits 3-phosphoinositide-dependent protein kinase 1 (PDK1) *in vitro* (IC_50_ in the low nanomolar range) and the PDK1-dependent phosphorylation of Akt in cell lines and excised tumours. It is interesting to note that 2-*O*-Bn-InsP_5_ also inhibits the mammalian target of rapamycin (mTOR) *in vitro*.

**Conclusions:**

InsP_5_ and 2-*O*-Bn-InsP_5_ may represent lead compounds to develop novel inhibitors of the PI3K/Akt pathway (including potential dual PDK1/mTOR inhibitors) and novel potential anti-cancer drugs.

Phosphoinositide 3-kinase (PI3K) isoforms catalyse the phosphorylation of the 3-hydroxyl group within the inositol ring of phosphoinositides generating lipid products, which in turn mediate the activation of several proteins (Maffucci and Falasca, 2001; Vanhaesebroeck *et al*, 2001). The best characterised PI3K effector is the Serine/Threonine kinase protein kinase B (PKB)/Akt, which regulates a plethora of intracellular processes, including cell survival, growth, proliferation, migration and regulation of cell size (Vivanco and Sawyers, 2002; Manning and Cantley, 2007). Upon PI3K activation, interaction between Akt pleckstrin homology (PH) domain and the PI3K product phosphatidylinositol 3,4,5-trisphosphate (PtdIns(3,4,5)P_3_) recruits Akt to the plasma membrane, where it is activated through phosphorylation at its residues Thr308 and Ser473. Phosphorylation of Thr308 is mediated by 3-phosphoinositide-dependent protein kinase 1 (PDK1), which itself possesses a PH domain able to bind PtdIns(3,4,5)P_3_ (Komander *et al*, 2004). Mutations in PDK1 PH domain that abolish PtdIns(3,4,5)P_3_ binding strongly inhibit Akt activation in homozygous knock-in embryonic stem cell and knock-in mice (McManus *et al*, 2004; Bayascas *et al*, 2008). On the other hand, binding of Akt PH domain to PtdIns(3,4,5)P_3_ is critical to induce a conformational change that allows PDK1-dependent phosphorylation (Calleja *et al*, 2007). Among other targets, Akt activates the multi-protein complex mTORC1 containing the enzyme mammalian target of rapamycin (mTOR), which regulates several intracellular functions including cell growth, cell cycle progression and autophagy (Wullschleger *et al*, 2006). It is interesting to note that a second mTOR-containing complex (mTORC2) is involved in the phosphorylation of Akt at its residue Ser473 as well as its activation (Sarbassov *et al*, 2005). The mechanism of mTORC2-dependent Akt phosphorylation at Ser473 is still not completely understood, but it does not seem to involve phosphoinositides, as in the case of PDK1, since mTOR does not appear to possess phosphoinositide-binding domains.

Deregulation of PI3K-dependent signalling pathways is linked to the development of cancer (Maehama and Dixon, 1999; Shayesteh *et al*, 1999; Vivanco and Sawyers, 2002; Bader *et al*, 2005; Shaw and Cantley, 2006; Vogt *et al*, 2007) and to increased resistance to treatment with chemotherapeutic agents (Clark *et al*, 2002; Liang *et al*, 2003; She *et al*, 2003). Accumulation of PtdIns(3,4,5)P_3_ either due to gain of function of PI3K activity (Vivanco and Sawyers, 2002; Vogt *et al*, 2007) or loss of the enzyme phosphatase and tensin homolog deleted on chromosome 10 (PTEN), which specifically dephosphorylates PtdIns(3,4,5)P_3_ (Maehama and Dixon, 1999), has been detected in almost 50% of all tumour types (Carracedo and Pandolfi, 2008). Reducing the levels of PDK1 in PTEN^+/−^ mice strongly protects them from developing a wide range of tumours (Bayascas *et al*, 2005). Furthermore, it has recently been reported that PDK1 is overexpressed in several human breast cancers and that increased copy number of the gene encoding for PDK1 is associated with upstream pathway lesions and patient survival (Maurer *et al*, 2009), highlighting the importance of PDK1 in cancer development. Elevated Akt activity has been found in several forms of cancer (Sun *et al*, 2001; Bacus *et al*, 2002; Altomare *et al*, 2004) and evidence suggest that mTORC1 is one of the key effectors in PI3K/Akt-mediated tumourigenesis (Guertin and Sabatini, 2007). The crucial role of mTORC2 in tumorigenesis driven by *Pten* loss has also been reported (Guertin *et al*, 2009). The PI3K/Akt pathway is, therefore, at present being considered to be an attractive target for therapeutic intervention, and several compounds targeting the different components of the pathway have been developed or are in development (Vivanco and Sawyers, 2002; Luo *et al*, 2003; Hennessy *et al*, 2005; Guertin and Sabatini, 2007; Liu *et al*, 2009), with some of them currently in clinical trials for cancer treatment (Liu *et al*, 2009). Toxicity, low therapeutic index, insolubility and aqueous instability have prevented the use of generic PI3K inhibitors wortmannin and LY294002 as anti-cancer agents despite their anti-tumour activity (Hu *et al*, 2000; Ng *et al*, 2001). More specific approaches are needed to selectively block only the deregulated rather than all the PI3Ks-dependent pathways. Small molecule inhibitors of PDK1 have recently been developed, most of which target the ATP-binding site of PDK1, but they often possess poor physicochemical properties and inadequate selectivity profiles (Peifer and Alessi, 2008). Similarly several Akt inhibitors have been designed including compounds targeting its ATP-binding domain or allosteric inhibitors and pseudosubstrates (Luo *et al*, 2005; Crowell *et al*, 2007; Lindsley *et al*, 2008). However, some of these agents show toxic side effects either because of non-specific effects or blockade of all Akt isoforms, thus resulting in the alteration of normal glucose homeostasis. The *in vitro* and *in vivo* effects on Akt of chemopreventive compounds, such as the rotenoid deguelin have also been reported (Lee *et al*, 2005). Finally, several mTOR inhibitors are at present available, whose effects have been investigated in many solid tumours (Guertin and Sabatini, 2007; Fasolo and Sessa, 2008). Despite several efforts, there is still a need to develop novel, more potent inhibitors of the PI3K/Akt pathway to overcome problems of lack of specificity and chemoresistance.

A few years ago we were the first to propose an alternative mechanism to block Akt activation based on the inhibition of its PH domain-mediated translocation to the plasma membrane (Berrie and Falasca, 2000). The critical role of the PH domain in Akt-driven tumourigenesis has recently been highlighted by the detection of a somatic mutation in Akt1 PH domain resulting in Akt1 activation in breast, colorectal and ovarian cancers (Carpten *et al*, 2007). It is interesting to note that this mutant is able to induce leukaemia in mice (Carpten *et al*, 2007). Our strategy was based on the hypothesis that specific exogenous inositol polyphosphates can compete with PtdIns(3,4,5)P_3_ by binding to Akt PH domain, and thus prevent recruitment to the plasma membrane and activation of Akt (Berrie and Falasca, 2000). Indeed we reported that inositol 1,3,4,5,6-pentakisphosphate (InsP_5_) specifically blocks Akt activation and possesses pro-apoptotic (Razzini *et al*, 2000; Piccolo *et al*, 2004), anti-angiogenic and anti-tumour activity *in vivo* (Maffucci *et al*, 2005). In addition, some of us recently demonstrated the targeting of the Akt PH domain with an unusual inositol polyphosphate mimic (Mills *et al*, 2007). Other phosphatidylinositol-based Akt inhibitors also act by inhibiting Akt targeting to the plasma membrane, including ether lipid analogues and PH domain-targeting inhibitors (Kozikowski *et al*, 2003; Gills *et al*, 2006; Crowell *et al*, 2007) such as perifosine, the most developed Akt inhibitor currently available (Kondapaka *et al*, 2003).

In order to explore early structure-activity relationships for InsP_5_ and possibly obtain more potent and specific inhibitors of the PI3K/Akt pathway, we synthesised novel compounds based on the InsP_5_ structure. Here we show that the derivative 2-*O*-benzyl-*myo*-inositol 1,3,4,5,6-pentakisphosphate (2-*O*-Bn-InsP_5_) exhibits more efficient and potent activity than InsP_5_ not only in inhibiting Akt phosphorylation but also in inducing apoptosis in different human cancer cell lines. It is interesting to note that 2-*O*-Bn-InsP_5_ promotes apoptosis in cell lines normally resistant to treatment with InsP_5_ and markedly inhibits the *in vivo* growth of InsP_5_-resistant xenografts. Kinase profiling analysis reveals that 2-*O*-Bn-InsP_5_ strongly inhibits PDK1 activity *in vitro* with an IC_50_ in the low nanomolar range. This is mirrored by the inhibition of Akt phosphorylation at its residue Thr308 in 2-*O*-Bn-InsP_5_-treated cells and in tumours from 2-*O*-Bn-InsP_5_-treated mice. Furthermore, the effect of 2-*O*-Bn-InsP_5_ is highly specific, as this compound only inhibits PDK1 and to a lesser extent mTOR in a panel of almost 60 kinases. These data represent the first attempt to exploit InsP_5_ as a potential lead compound for the development of potent small molecule inhibitors of the PI3K/Akt pathway.

## Materials and methods

### Materials

Inositol 1,3,4,5,6-pentakisphosphate was synthesised as previously reported (Godage *et al*, 2006). 2-*O*-Bn-InsP_5_ was synthesised in a similar manner from 2-*O*-benzyl-*myo*-inositol. Each compound was purified to homogeneity by ion-exchange chromatography on Q-Sepharose Fast Flow resin (GE Healthcare Life Sciences, Little Chalfont, Buckinghamshire, UK) and used as the triethylammonium salt, which was fully characterized by ^31^P and ^1^H spectroscopy and accurately quantified by total phosphate assay. For the *in vivo* experiments, InsP_5_ and 2-*O*-Bn-InsP_5_ were each converted into the hexasodium salt by treatment with Dowex 50WX2-100 ion-exchange resin (Sigma-Aldrich, Gillingham, Dorset, UK), followed by addition of sodium hydroxide (6 equivalents) and lyophilisation. Sulphorhodamine (SRB), curcumin, paclitaxel and 4-hydroxy-tamoxifen were purchased from Sigma-Aldrich; anti-phospho Ser473 Akt, anti-phospho Thr308 Akt and anti-Akt from (Cell Signaling Technologies, Danvers, MA, USA) or Santa Cruz Biotechnology (Santa Cruz, CA, USA).

### Cell lines

SKOV-3 and PC3 were cultured in RPMI 1640; all other cell lines were cultured in DMEM. Media were supplemented with 10% FBS, penicillin/streptomycin and glutamine.

### Cell survival and apoptosis assays

Cells seeded in a 24-well plate were treated with the indicated compounds in serum free DMEM or DMEM containing 0.5% FBS (PC3). After 72 h, the number of surviving cells was assessed by manual cell counting or by using the cell counter CDA-500 (Sysmex, Milton Keynes, UK). Alternatively, after 48 h, the number of apoptotic cells was assessed by acridine orange/ethidium bromide assay as described (Piccolo *et al*, 2004; Maffucci *et al*, 2005). SRB test was carried out in SKOV-3 and PC3 seeded in 96-well plate (3800 cells per well or 5800 cells per well, respectively) after 72 h of treatment as described (Cappella *et al*, 2001).

### *In vivo* studies

Male nude athymic CD-1 nu/nu mice (8-weeks old) were obtained from Harlan (San Pietro al Natisone, Italy) and maintained under specific pathogen-free conditions with food and water provided *ad libitum*. The general health status of the animals was monitored daily. Procedure involving animals and their care were conducted in conformity with the institutional guidelines that are in compliance with national and international laws and policies.

### Toxicity assay

Male nude CD-1 mice were treated with a single dose of 750 mg kg^−1^ InsP_5_ or 2-*O*-Bn-InsP_5_/mouse administered intraperitoneally (i.p.). Each group consisted of 2–3 mice. Body weight, deaths and any other sign of toxicity and changes in behaviour (such as motility, eating and drinking habits) were recorded.

### Anti-tumour activity assay

Exponentially growing PC3 cells were harvested, washed twice and resuspended in PBS at a concentration of 2.5 × 10^7^ cells ml^−1^. A suspension of 5 × 10^6^ PC3 cells was injected subcutaneously (s.c.) into the left flank of the recipient mice. When tumours reached a size of ∼70 mm^3^ (approximately 15 days after tumour cell implant), mice were divided into seven groups (*n*=7). InsP_5_ and 2-*O*-Bn-InsP_5_ were administered by daily i.p. injections at different doses of 12.5–25–50 mg kg^−1^ day^-1^ for 14 consecutive days. Control mice were treated with water in an equal volume. The diameters of s.c. growing tumours were measured with a caliper twice a week and the experiment was ended at day 28 after the implantation.

### Data analysis and *in vivo* tumour parameters

The volume of s.c. growing tumours was calculated by the formula: Tumour weight (mg)=(length × width^2^)/2. Differences in s.c tumour growth between the treatment groups were evaluated with a one-way ANOVA followed by Fisher's test using the StatView statistical package (SAS Institute, Cary, NC, USA). The percentage of tumour growth was calculated as T/C%=(RTV-treated animals/RTV-control animals) × 100, where RTV was the mean relative tumour volume calculated as RTV=*V*_t_/*V*_0_. *V*_t_ was the tumour volume on the day of measurement and *V*_0_ was the tumour volume at the beginning of the treatment. The percentage of tumour weight inhibition (TWI%) was calculated using the formula: TWI%=100–T/C%. The log cell kill (LCK) was calculated using the formula: LCK=*T*−*C*/3.32 × *Td*, where *T−C* is the tumour growth delay calculated as the difference in median time (in days) required for the tumours in the treatment (*T*) and control group (*C*) to reach a predetermined size (i.e., 1000 mg). *Td* is the tumour volume doubling time in days, determined in the exponential growth phase of the control group from a best-fit straight line. Median doubling time was 3 days in control animals.

### Western blot

Mice with s.c. growing tumours were treated with a single dose of InsP_5_ and 2-*O*-Bn-InsP_5_ (50 mg kg^−1^) or vehicle. Animals were killed 24 h after treatment and tumour samples were collected and snap frozen. Frozen specimens of tumour tissue were homogenised with a Polytron homogeniser in a lysis buffer (ratio 1 : 1 w/v) containing 50 mM Tris-HCl (pH 7.4), 5 mM EDTA, 0.1% Nonidet NP-40, 250 mM NaCl, 50 mM NaF and proteases and phosphatase inhibitors. After centrifugation at 13 000 r.p.m. for 10 min at 4°C, 80 μg of protein was separated on SDS–PAGE and transferred to a polyvinylidene difluoride membrane (Millipore, MA, USA). Membranes were probed with the indicated antibodies.

### Protein kinase profiling

Effect of the indicated compounds on the activity of various kinases was assessed by SelectScreen Kinase Profiling Service (Invitrogen-Life Technologies, Paisley, UK). Assays were performed using 1 *μ*M of the tested compounds and ATP concentration as indicated in the corresponding tables. In the case of InsP_5_ a screen using 10 *μ*M of the compound was also carried out, as indicated in the corresponding table.

## Results

### Synthesis of novel potential inhibitors of the PI3K/Akt pathway and *in vitro* screening

**Figure 1:**
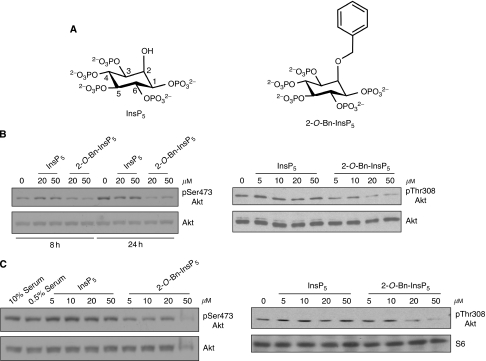
*In vitro* activity of inositol 1,3,4,5,6-pentakisphosphate (InsP_5_) and 2-*O*-benzyl-*myo*-inositol 1,3,4,5,6-pentakisphosphate (2-*O*-Bn-InsP_5_). (**A**) Structure of inositol 1,3,4,5,6-pentakisphosphate (InsP_5_) and 2-*O*-benzyl-*myo*-inositol 1,3,4,5,6-pentakisphosphate (2-*O*-Bn-InsP_5_). (**B**, **C**) SKOV-3 were treated for 8 h or 24 h with the indicated concentrations of InsP_5_ or 2-*O*-Bn-InsP_5_ in serum free medium, (**B**) while prostate cancer PC3 cells were treated for 24 h with the indicated concentrations of InsP_5_ or 2-*O*-Bn-InsP_5_ in medium containing 0.5% FBS (**C**). Akt activation was assessed by monitoring phosphorylation at its residues Ser473 and Thr308. Membranes were then stripped and re-probed with the indicated antibodies.

We have recently reported that InsP_5_ is a novel inhibitor of the PI3K/Akt pathway, which possesses pro-apoptotic, anti-angiogenic and anti-tumour activity (Razzini *et al*, 2000; Piccolo *et al*, 2004; Maffucci *et al*, 2005). To explore the design of novel inhibitors of the PI3K/Akt pathway, potentially more active than InsP_5_, we decided to modify the structures of either Ins(1,3,4,5)P_4_ or InsP_5_. Different strategies were used to modify the parent molecules and several compounds were synthesised and tested. For Ins(1,3,4,5)P_4_-related compounds, modifications were made at C-6 because X-ray structures of the Akt (Thomas *et al*, 2002) and PDK1 (Komander *et al*, 2004) PH domains in complex with Ins(1,3,4,5)P_4_ indicated that the 6-hydroxyl group of Ins(1,3,4,5)P_4_ is not directly involved in binding. Furthermore, the X-ray structure of Akt PH domain showed that a tyrosine residue near the 6-OH of bound Ins(1,3,4,5)P_4_ might interact with an aromatic group. In the case of InsP_5_ analogs, modifications were on either the 2-*O*-atom or the 5-phosphate, thus maintaining the symmetry of the parent molecule. The derivatives were first tested for their ability to inhibit Akt activation in cell lines characterised by constitutive activation of the PI3K/Akt pathway and with a reported sensitivity to InsP_5_, namely ovarian cancer cells SKOV-3 and breast cancer cells SKBR3 (Piccolo *et al*, 2004; Maffucci *et al*, 2005). In this original screening we observed that the InsP_5_ derivative 2-*O*-benzyl-*myo*-inositol 1,3,4,5,6-pentakisphosphate (Figure 1A, named 2-*O*-Bn-InsP_5_) showed the highest efficiency in inhibiting Akt activation in all cell lines tested (data on the other derivatives will be published elsewhere). More specifically, we found that 2-*O*-Bn-InsP_5_ inhibited Akt phosphorylation at its residue Ser473 more efficiently than InsP_5_ in SKOV-3, being already active after 8 h of treatment and at a concentration of 20 *μ*M (Figure 1B). Inhibition of Akt phosphorylation at residue Thr308 was also detected (Figure 1B). More interestingly, we found that 2-*O*-Bn-InsP_5_ was able to block Akt phosphorylation in cell lines resistant to InsP_5_, such as prostate cancer cells PC3 (Figure 1C) and pancreatic cancer cells ASPC1 (results not shown). Taken together these data indicate that structural modification at the C-2 of InsP_5_ can enhance its inhibitory properties towards Akt activation.

### Analysis of the biological activity of 2-*O*-Bn-InsP_5_

**Figure 2:**
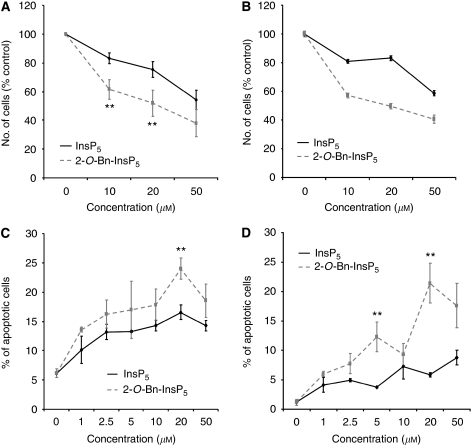
2-*O*-benzyl-*myo*-inositol 1,3,4,5,6-pentakisphosphate (2-*O*-Bn-InsP_5_) possesses higher pro-apoptotic activity than inositol 1,3,4,5,6-pentakisphosphate (InsP_5_). (**A**, **B**) SKBR3 (**A**) and SKOV-3 (**B**) were treated for 72 h with the indicated concentrations of InsP_5_ or 2-*O*-Bn-InsP_5_. The number of surviving cells was assessed by cell counting. Data are mean±s.e. of *n*=4 (**A**) and *n*=2 (**B**) independent experiments. ^**^=*P*<0.05. (**C**, **D**) SKBR3 (**C**) and SKOV-3 (**D**) were treated with the indicated concentrations of InsP_5_ or 2-*O*-Bn-InsP_5_. The number of apoptotic cells was assessed by acridine orange/ethidium bromide assay. Data are mean±s.e. of three independent experiments. ^**^=*P*<0.05.

**Figure 3:**
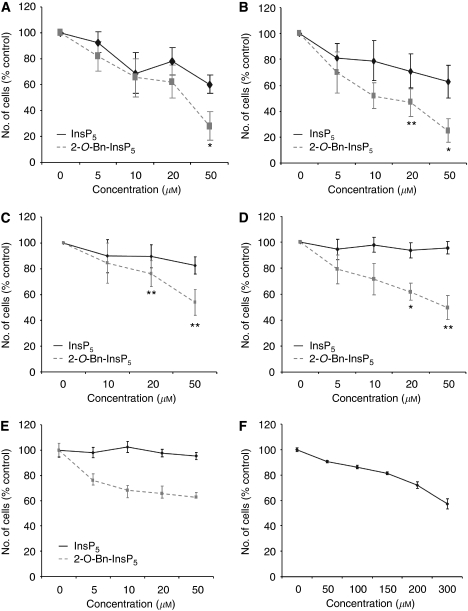
2-*O*-benzyl-*myo*-inositol 1,3,4,5,6-pentakisphosphate (2-*O*-Bn-InsP_5_) possesses pro-apoptotic activity in cell lines resistant to inositol 1,3,4,5,6-pentakisphosphate (InsP_5_). (**A**–**D**) BxPc-3 (**A**), ASPC1 (**B**), MDA-MB-468 (**C**) and PC3 (**D**) were treated for 72 h with the indicated concentrations of InsP_5_ or 2-*O*-Bn-InsP_5_. The number of surviving cells was assessed by cell counting. Data are mean±s.e. of *n*=3 (**A**), *n*=6 (**B**), *n*=3 (**C**) and *n*=4 (**D**) independent experiments carried out in duplicate. ^*^=*P*<0.01; ^**^=*P*<0.05. (**E**, **F**) PC3 were treated with the indicated concentrations of InsP_5_ and 2-*O*-Bn-InsP_5_ (**E**) or increasing concentrations of InsP_5_ (**F**). After 72 h the number of surviving cells was assessed by SRB assay. Data are mean±s.e. of *n*=2 independent experiments.

We next compared the effects of 2-*O*-Bn-InsP_5_ and InsP_5_ on proliferation/survival of cancer cells *in vitro*. Treatment with 2-*O*-Bn-InsP_5_ strongly reduced the number of surviving SKBR3 (Figure 2A) and SKOV-3 (Figure 2B) assessed by cell counting. In particular, 2-*O*-Bn-InsP_5_ was more active than InsP_5_ in both the cell lines. Acridine orange/ethidium bromide assay confirmed that the percentage of apoptotic cells was higher in 2-*O*-Bn-InsP_5_-treated compared with InsP_5_-treated SKBR3 (Figure 2C) and SKOV-3 (Figure 2D). Based on the data on Akt phosphorylation we then decided to analyse the effect of 2-*O*-Bn-InsP_5_ on the survival of cell lines normally very resistant to InsP_5_ treatment. 2-*O*-Bn-InsP_5_ was more potent than InsP_5_ at a concentration of 50 *μ*M in pancreatic cancer cells BxPc-3 (Figure 3A), whereas it was more active than InsP_5_ at almost all concentrations tested in pancreatic cancer cells ASPC1 (Figure 3B). A stronger activity of 2-*O*-Bn-InsP_5_ compared with InsP_5_ was also observed in breast cancer cells MDA-MB-468 (Figure 3C) and in PC3 (Figure 3D), consistent with data on Akt phosphorylation. Higher activity of 2-*O*-Bn-InsP_5_ in PC3 cells was also observed in SRB assays (Figure 3E). It is important to note that although InsP_5_ had no effect in PC3 at concentrations up to 50 *μ*M, concentrations of 200–300 *μ*M were eventually able to mimic the effect of 2-*O*-Bn-InsP_5_ in PC3 cells (Figure 3F), thus suggesting that 2-*O*-Bn-InsP_5_ is acting on the same intracellular pathway as InsP_5_. Taken together these data demonstrate that addition of a benzyl group to the axial 2-O atom of InsP_5_ potentiates the pro-apoptotic properties of the compound not only in cells sensitive to InsP_5_ but also in cells normally very resistant to treatment with the parent inositol compound.

### *In vivo* anti-tumour activity of 2-*O*-Bn-InsP_5_ on InsP_5_-resistant xenografts

**Figure 4:**
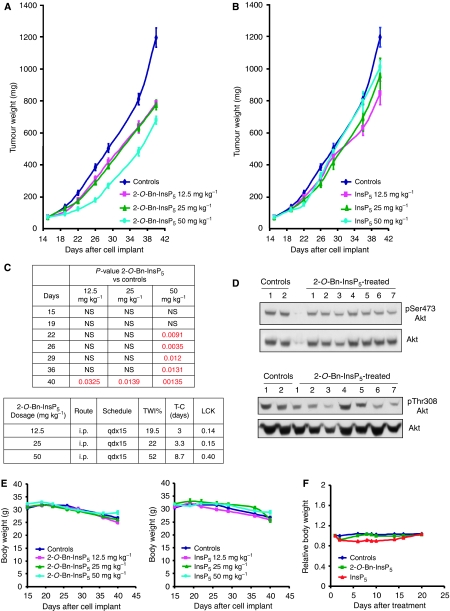
2-*O*-benzyl-*myo*-inositol 1,3,4,5,6-pentakisphosphate (2-*O*-Bn-InsP_5_) possesses anti-tumour activity on inositol 1,3,4,5,6-pentakisphosphate (InsP_5_)-resistant xenografts and it is not associated with toxicity *in vivo*. Male athymic CD-1 nu/nu mice were inoculated subcutaneously (s.c.) with PC3 and treated with the indicated concentrations of either InsP_5_ or 2-*O*-Bn-InsP_5_ from day 15 after cell implantation. The inositol compounds (12.5–25–50 mg kg^−1^ day^−1^) and vehicle (water) were given daily by intraperitoneal (i.p.) injections for 14 consecutive days (days 15–28). Tumour size was assessed twice weekly. (**A**, **B**) Tumour growth in 2-*O*-Bn-InsP_5_-treated mice (**A**) and InsP_5_-treated mice (**B**) compared with control group measured for the duration of the experiment. Results are expressed as mean±s.e. (**C**) Top: Table showing all *P*-values for the indicated doses of 2-*O*-Bn-InsP_5_ compared to controls at the indicated days of treatment (NS=not significant). Bottom: *In vivo* anti-tumour activity parameters. The percentage of tumour weight inhibition (TWI%), the tumour growth delay (T−C) and the log cell kill (LCK) were calculated as described in the MATERIALS AND METHODS section. The highest inhibition of tumour volume is reported. (**D**) Mice with s.c. growing tumours were treated with a single dose (50 mg kg^−1^) of InsP_5_, 2-*O*-Bn-InsP_5_ or water (control). Tumours were excised 24 h after treatment. Phosphorylation of Akt at its residues Ser473 and Thr308 was assessed by using specific antibodies. Membranes were then stripped and re-probed with an anti-Akt antibody. (**E**) Body weights of mice treated with 2-*O*-Bn-InsP_5_ or InsP_5_ for 14 consecutive days. (**F**) Body weights of mice treated with a single dose of 750 mg kg^−1^ InsP_5_ or 2-*O*-Bn-InsP_5_.

We then decided to test the therapeutic efficacy of 2-*O*-Bn-InsP_5_ in human tumour xenografts characterised by the activation of PI3K/Akt pathway and higher sensitivity to 2-*O*-Bn-InsP_5_ compared with InsP_5_. We specifically implanted PC3 cells in nude mice and 15 days after the implantation we treated groups of mice with different concentrations (12.5, 25 and 50 mg kg^−1^) of InsP_5_ or 2-*O*-Bn-InsP_5_ for 14 consecutive days (from day 15 to day 28). Tumour growth was followed for further 12 days after the end of the treatment (upto day 40). Data revealed that 2-*O*-Bn-InsP_5_ at doses of 12.5 and 25 mg kg^−1^ clearly decreased the growth of tumours compared with untreated mice, although the differences were statistically significant only on the last day of measurement (Figure 4A and C). A strong reduction in tumour growth was obtained in the group treated with 50 mg kg^−1^ 2-*O*-Bn-InsP_5_, with a statistically significant difference *vs* controls detectable from day 22 after tumour cells implant onwards (Figure 4A and C). Data on *in vivo* anti-tumour activity parameters relative to 2-*O*-Bn-InsP_5_ are shown in Figure 4C, bottom table. More than 50% inhibition of tumour weight was achieved in the 50 mg kg^−1^-treated group, with a tumour growth delay (T−C) of almost 9 days between this group and the untreated (control) group. In agreement with our *in vitro* data, we observed that InsP_5_ had no effect on concentrations up to 50 mg kg^−1^ (Figure 4B). At the end of the experiment, western blot analysis revealed that 24 h-treatment with 2-*O*-Bn-InsP_5_ markedly reduced Akt phosphorylation at its residue Ser473 in all 2-*O*-Bn-InsP_5_-treated mice (Figure 4D). Furthermore, a clear inhibition of Akt phosphorylation at its residue Thr308 was detected in five out of seven 2-*O*-Bn-InsP_5_-treated mice (Figure 4D). It is noteworthy that no evidence of toxicity was observed in different groups of mice at all the tested doses of either 2-*O*-Bn-InsP_5_ or InsP_5_ and the body weight of the treated animals was not different from the untreated mice throughout the entire experiment (Figure 4E). Moreover, a single treatment with a very high dose of 2-*O*-Bn-InsP_5_ or InsP_5_ (750 mg kg^−1^) did not cause any major toxic effect (Figure 4F). Taken together these data demonstrate that 2-*O*-Bn-InsP_5_ is able to inhibit growth of InsP_5_-resistant tumours through a more efficient blockade of Akt phosphorylation *in vivo*.

### *In vitro* kinase profiling of InsP_5_ and 2-*O*-Bn-InsP_5_

To determine the mechanism responsible for the higher activity of 2-*O*-Bn-InsP_5_, we decided to carry out a protein kinase activity screen for InsP_5_ and 2-*O*-Bn-InsP_5_ (SelectScreen Kinase Profiling Service, Invitrogen-Life Technologies). Among almost 60 protein kinases screened, 2-*O*-Bn-InsP_5_ (1 *μ*M) showed a very high inhibitory activity towards PDK1 (79% inhibition) and a lower activity towards mTOR (Table 1A, Supplementary Table 1). 2-*O*-Bn-InsP_5_ did not inhibit (percentage of inhibition <40%) any of all the other tested kinases, including AGC kinases, such as GSK3, RSK, S6K and members of the PKC family, AMPK and several members of the MAPK family (Supplementary Table 1). Furthermore 2-*O*-Bn-InsP_5_ did not directly inhibit any of the class I PI3K isoforms tested or any Akt isoforms (Table 1A, Supplementary Table 1). InsP_5_ showed a reduced inhibitory effect on PDK1 compared with 2-*O*-Bn-InsP_5_ (Table 1B and C, Supplementary Table 2 and 3). As 2-*O*-Bn-InsP_5_, when tested on a panel of over 50 kinases and at a concentration of 10 *μ*M, InsP_5_ did not significantly inhibit any of the tested kinases (Supplementary Table 3) including Akt isoforms (Table 1B). In contrast to 2-*O*-Bn-InsP_5_, InsP_5_ did not inhibit mTOR, even when tested at a concentration of 10 *μ*M (Supplementary Table 3). Comparing the effect of 1 μM of different natural inositol polyphosphates on PDK1, InsP_5_ possessed the highest inhibitory activity towards PDK1 (71% inhibition) with only Ins(1,3,4,5)P_4_ also showing some effect (56% inhibition). None of the other polyphosphates had any significant effect (Table 1C). These data indicate that 2-*O*-Bn-InsP_5_ and InsP_5_ inhibit PDK1 very specifically, with 2-*O*-Bn-InsP_5_ possessing the highest inhibitory activity towards PDK1. Indeed results from SelectScreen Kinase Profiling Service (Invitrogen-Life Technologies) 10-point titration revealed that the IC_50_ of InsP_5_ towards PDK1 was 613 nM whereas the corresponding IC_50_ of 2-*O*-Bn-InsP_5_ was a striking 26.5 nM (Table 1A and B). These data clearly indicate that 2-*O*-Bn-InsP_5_ is a novel, potent and highly selective PDK1 inhibitor. This is consistent with the detected inhibition of Thr308 phosphorylation in 2-*O*-Bn-InsP_5_-treated SKOV-3 and PC3 cells (Figure 1B and C) and 2-O-Bn-InsP_5_-treated mice (Figure 4D). Furthermore, 2-*O*-Bn-InsP_5_, but not InsP_5_, is able to inhibit mTOR selectively *in vitro* with an IC_50_ of 1.3 *μ*M (Table 1A).

### *In vitro* effects of 2-*O*-Bn-InsP_5_ in combination with anti-cancer compounds

**Figure 5:**
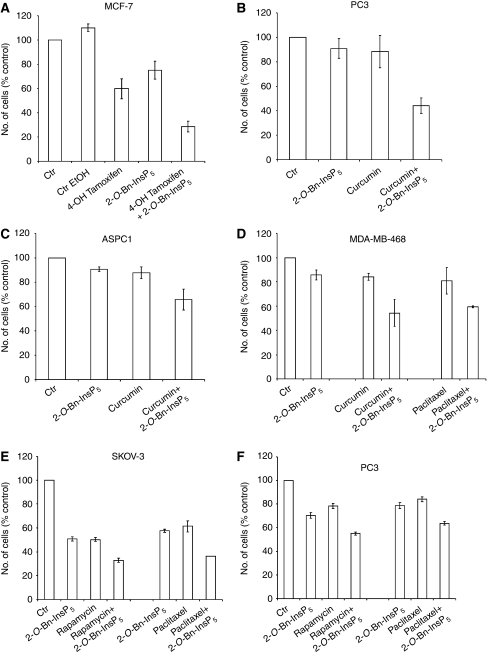
Combination of 2-*O*-benzyl-*myo*-inositol 1,3,4,5,6-pentakisphosphate (2-*O*-Bn-InsP_5_) with anti-cancer compounds *in vitro* results in additive or more than additive effects. (**A**) MCF7 were treated with 20 nM 4-OH Tamoxifen, 50 *μ*M 2-*O*-Bn-InsP_5_ alone or in combination. Data are mean±s.e. of *n*=4 independent experiments carried out in duplicate. 4-OH Tamoxifen+2-*O*-Bn-InsP_5_: *P*<0.01 *vs* 4-OH Tamoxifen; *P*<0.01 *vs* 2-*O*-Bn-InsP_5_. (**B**) PC3 were treated with 5 *μ*M 2-*O*-Bn-InsP_5_, 10 *μ*M curcumin alone or in combination. Data are mean±s.e. of *n*=5 independent experiments carried out in duplicate. Curcumin+2-*O*-Bn-InsP_5_: *P*<0.01 *vs* 2-*O*-Bn-InsP_5_; *P*<0.05 *vs* curcumin. (**C**) ASPC1 were treated with 5 *μ*M 2-*O*-Bn-InsP_5_, 10 *μ*M curcumin alone or in combination. Data are mean±s.e. of *n*=6 independent experiments carried out in duplicate. Curcumin+2-*O*-Bn-InsP_5_: *P*<0.05 *vs* 2-*O*-Bn-InsP_5_; *P*<0.05 *vs* curcumin. (**D**) MDA-MB-468 were treated with 10 *μ*M 2-*O*-Bn-InsP_5_, 10 *μ*M curcumin alone, 1 nM paclitaxel or the indicated combination. Data are mean±s.e. of *n*=2 independent experiments carried out in duplicate. In all cases (**A**–**D**), cells were treated for 72 h and the number of surviving cells was assessed by cell counting. (**E**) SKOV-3 were treated with 20 *μ*M 2-*O*-Bn-InsP_5_, 30 nM paclitaxel, 20 nM rapamycin or with the indicated combinations. (**F**) PC3 were treated with 10 *μ*M 2-*O*-Bn-InsP_5_, 1 nM rapamycin alone or in combination and with 20 *μ*M 2-*O*-Bn-InsP_5_, 40 nM paclitaxel alone or in combination. In all cases (**E**, **F**), cells were treated for 72 h and the number of surviving cells was assessed by SRB assay and data are mean±s.e. of two experiments carried out in quadruplicate.

Parallel RNAi and compound screens have recently revealed that PDK1 is a critical determinant of sensitivity to tamoxifen in breast cancer cells MCF7 (Iorns *et al*, 2009). Based on this result we decided to investigate whether inhibition of PDK1 by 2-*O*-Bn-InsP_5_ was able to sensitise MCF7 to the pro-apoptotic effect of tamoxifen. Our data revealed that treatment with 4-OH tamoxifen (the active metabolite of tamoxifen) for 72 h reduced the number of surviving cells, whereas 2-*O*-Bn-InsP_5_ had little effect (Figure 5A). It is interesting to note that the combination of 2-*O*-Bn-InsP_5_ and 4-OH tamoxifen strongly enhanced the effect of 4-OH tamoxifen or 2-*O*-Bn-InsP_5_ alone (Figure 5A). We then tested the effects of 2-*O*-Bn-InsP_5_ in combination with several natural anti-cancer compounds. The concentrations of the different compounds used in these experiments were minimally effective based on preliminary dose-response experiments (results not shown). A combination of 2-*O*-Bn-InsP_5_ and curcumin, a component of turmeric (*Curcuma longa*), strongly reduced the number of surviving PC3 cells, resulting in a more than additive effect (Figure 5B) and was able to enhance the effect of curcumin in ASPC1 (Figure 5C) and in MDA-MB-468 (Figure 5D). A combination of 2-*O*-Bn-InsP_5_ and paclitaxel clearly reduced the number of surviving MDA-MB-468 (Figure 5D), SKOV-3 (Figure 5E) and PC3 (Figure 5F) cells compared with the corresponding single treatments. An additive effect was detected when combining 2-*O*-Bn-InsP_5_ with rapamycin in SKOV-3 (Figure 5E) and PC3 (Figure 5F) cells. These data clearly indicate that the combination of 2-*O*-Bn-InsP_5_ and natural anti-cancer compounds results in additive or more than additive effects, and therefore suggest that 2-*O*-Bn-InsP_5_ can potentially be used in combination with natural compounds to increase their anti-cancer activity.

## Discussion

The *in vivo* anti-tumour activity of InsP_5_ together with the lack of toxicity observed using this compound (Maffucci *et al*, 2005), suggested that InsP_5_ might represent a lead compound to design novel inhibitors of the PI3K/Akt pathway to be eventually brought into clinical testing. InsP_5_ possesses very few sites for chemical modification, the axial 2-hydroxyl group being the most realistic possibility. Here we describe one InsP_5_ derivative, 2-*O*-Bn-InsP_5_, which possesses enhanced pro-apoptotic and anti-tumour activity compared with the parent molecule. In this respect 2-*O*-Bn-InsP_5_ represents a first step towards the development of novel efficient anti-cancer drugs targeting the PI3K/Akt pathway and based on the InsP_5_ structure. Kinase profiling assays revealed that 2-*O*-Bn-InsP_5_ potently and specifically inhibits PDK1 *in vitro* and the PDK1-dependent phosphorylation of Thr308 Akt in cell lines and *in vivo*. These results are particularly important considering that, to our knowledge, no specific and selective PDK1 inhibitors are at present available and they make 2-*O*-Bn-InsP_5_ an interesting new molecule to use as a model for designing novel specific PDK1 inhibitors. In addition 2-*O*-Bn-InsP_5_ is able to inhibit mTOR at least *in vitro* (albeit to a lesser extent than PDK1). It is noteworthy that PDK1 and mTOR were the only enzymes to be inhibited by 2-*O*-Bn-InsP_5_ in a screen of almost 60 different kinases, indicating that 2-*O*-Bn-InsP_5_ may represent an interesting lead compound to design novel and potent dual PDK1 and mTOR inhibitors.

2-*O*-benzyl-*myo*-inositol 1,3,4,5,6-pentakisphosphate was selected in a screen of several different compounds that we synthesised and first tested for their ability to inhibit Akt phosphorylation in cells sensitive to InsP_5_. In this original screen, 2-*O*-Bn-InsP_5_ was not only more efficient than the parent molecule in inhibiting Akt phosphorylation and inducing apoptosis in InsP_5_-sensitive cell lines but it was also able to induce apoptosis in InsP_5_-resistant cell lines including pancreatic cancer cells. The pro-apoptotic activity of 2-*O*-Bn-InsP_5_ detected in pancreatic cancer cells represents an extremely important result taking into account the high resistance of this cancer to chemotherapeutic treatment and the urgent need for novel therapeutics in clinical treatment. Furthermore, 2-*O*-Bn-InsP_5_ was able to inhibit the *in vivo* growth of InsP_5_-resistant prostate cancer xenografts. It is noteworthy that, although 2-*O*-Bn-InsP_5_ acted in the micromolar range in *in vitro* studies, it was able to inhibit tumour growth *in vivo* at 12.5, 25 and 50 mg kg^−1^, doses commonly used to test the *in vivo* effect of potential anti-tumour compounds. In particular, 2-*O*-Bn-InsP_5_ induced a tumour weight inhibition of 52% in prostate cancer xenografts when dosed at 50 mg kg^−1^ once daily for 14 days.

We then decided to investigate in more detail the mechanisms of action of InsP_5_ and 2-*O*-Bn-InsP_5_, and to explain the higher activity of 2-*O*-Bn-InsP_5_ compared with the parent molecule. Our previous and current data demonstrated that the *in vitro* and *in vivo* properties of InsP_5_ and 2-*O*-Bn-InsP_5_ were because of reduced Akt phosphorylation and activation (Piccolo *et al*, 2004; Maffucci *et al*, 2005). Results from the kinase profiling assays here show that InsP_5_ and 2-*O*-Bn-InsP_5_ do not inhibit Akt kinase activity itself *in vitro*, whereas they are both able to directly inhibit PDK1 kinase activity (albeit with different potency), thus indicating that the detected Akt inhibition is due to blockade of the activity of its upstream regulatory kinase. This is consistent with the observed enhanced activity of 2-*O*-Bn-InsP_5_ compared with InsP_5_, likely due to its higher inhibitory activity towards PDK1. Furthermore, this difference could explain the strong effect of 2-*O*-Bn-InsP_5_ in InsP_5_-resistant cell lines such as the PTEN mutant cells MDA-MB-468 and PC3, and would be consistent with the proposed key role of PDK1 in tumourigenesis driven by *Pten* loss (Bayascas *et al*, 2005). It should be noted that the resistance to InsP_5_ treatment in these cells is consistent with the delayed onset of inhibition in cells with mutant PTEN observed using the ether lipid analogues (Castillo *et al*, 2004). The observation that higher concentrations of InsP_5_ are eventually able to mimic the pro-apoptotic effect of 2-*O*-Bn-InsP_5_ in these cells further supports the conclusion that the different activity is because of different potency of the two compounds towards PDK1. In this respect, it is interesting to notice that the addition of the benzyl group to InsP_5_ confers such a higher inhibitory activity towards PDK1 on the derivative 2-*O*-Bn-InsP_5_. It would be interesting to investigate whether the resulting small increase in the hydrophobicity of the molecule enhances its activity possibly by improving its binding to PDK1. Moreover, it is noteworthy that such a modification confers to 2-*O*-Bn-InsP_5_ a selective inhibitory activity towards mTOR *in vitro*, providing crucial information to develop novel specific dual PI3K/mTOR inhibitors. Indeed one intriguing possibility is that 2-*O*-Bn-InsP_5_ is more active than InsP_5_ because of its unique capability to inhibit simultaneously and very specifically PDK1 and mTOR. In particular the dual activity of 2-*O*-Bn-InsP_5_ can explain its effect in prostate cancer cells PC3, consistent with the recently reported key role of mTORC2 in the development of loss of *Pten*-driven prostate cancer (Guertin *et al*, 2009). Furthermore, a potential 2-*O*-Bn-InsP_5_-mediated inhibition of mTORC2 would also increase the inhibitory activity towards Akt, by preventing its Ser473 phosphorylation, indicating that 2-*O*-Bn-InsP_5_ represents a useful compound to be tested in cancer types specifically dependent on mTOR activation. Studies have recently revealed the existence of a negative-feedback loop by which mTORC1 inhibition leads to upregulation of Akt (Manning, 2004; O’Reilly *et al*, 2006) and/or ERK/MAPK pathway (Carracedo *et al*, 2008), activating proliferative and anti-apoptotic signals in certain cancer types. The possibility of blocking PDK1 and mTOR simultaneously in these tumours is likely to be very effective therefore, because of its dual inhibitory activity towards both enzymes, it would be interesting to investigate the effect of 2-*O*-Bn-InsP_5_ in these cellular contexts.

Our future strategies to design novel compounds will take into consideration the possibility that, besides its direct inhibitory activity towards PDK1 and possibly mTOR, binding of 2-*O*-Bn-InsP_5_ to non-catalytic domains may affect the activity of kinases or their mechanism of activation *in vivo*. Indeed in our previous work we proposed that InsP_5_ can inhibit Akt activation by binding to Akt PH domain and preventing Akt recruitment to the plasma membrane (Berrie and Falasca, 2000). It was also proposed that inositol phosphates can bind PDK1 PH domain and retain this kinase in the cytosol, preventing Akt phosphorylation at Thr308 (Komander *et al*, 2004). These data suggest that the detected inhibitory effect of both 2-*O*-Bn-InsP_5_ and InsP_5_ on Akt activation *in vivo* can result from a combination of a direct effect on PDK1 kinase activity and effect on Akt/PDK1 recruitment to the plasma membrane. Similarly, the possibility that *in vivo* the inositol polyphosphates can bind and increase the activity of phosphatases which regulate Akt, such as PH domain leucine-rich repeat protein phosphatases 1 and 2 (Gao *et al*, 2005; Brognard *et al*, 2007) will be taken into consideration in our future strategies. In this respect it would be interesting to develop binding experiments of cellular lysates to immobilized 2-*O*-Bn-InsP_5_ and InsP_5_ to determine whether the compounds only bind PDK1, as indicated by the kinase profiling assays, or the *in vivo* mechanisms of action is more complex. These experiments would also give more information of whether the compounds may indirectly act on other kinases without directly affecting their catalytic activity.

It must be noted that, like InsP_5_, 2-*O*-Bn-InsP_5_ is a water soluble compound and it is well tolerated *in vivo* even at concentrations 15 times higher the active dose. In addition, combination of 2-*O*-Bn-InsP_5_ with other anti-cancer compounds including natural compounds results in additive or more than additive effects, indicating that such a compound (or derivatives) may prove particularly useful in combinatorial therapies. In particular 2-*O*-Bn-InsP_5_ increases the effect of tamoxifen in breast cancer cells MCF7, consistent with the reported role of PDK1 inhibition in tamoxifen sensitisation (Iorns *et al*, 2009). It is worth mentioning that our *in vitro* assays revealed that InsP_5_ itself is able to inhibit PDK1 (although less than 2-*O*-Bn-InsP_5_). This raises the interesting possibility that the endogenous intracellular InsP_5_ may act as an endogenous PDK1 inhibitor and regulator of the PI3K/Akt pathway. This hypothesis is currently being investigated in our laboratory.

In conclusion, here we have described the *in vitro* and *in vivo* properties of 2-*O*-Bn-InsP_5_, a derivative of InsP_5_, which possesses similar solubility and lack of toxicity *in vivo* but enhanced pro-apoptotic and anti-tumour activity compared with the parent molecule. In particular 2-*O*-Bn-InsP_5_ possesses specific inhibitory activity towards PDK1. Data also indicate that 2-*O*-Bn-InsP_5_ can inhibit mTOR, at least *in vitro*. It is interesting to note that InsP_5_ does not possess such an inhibitory activity towards mTOR, thus suggesting that comparison of the two molecules can give useful information towards developing specific dual PDK1/mTOR inhibitors. Taken together these data indicate that InsP_5_ and 2-*O*-Bn-InsP_5_ may represent promising models for further development of novel anti-cancer drugs.

## Figures and Tables

**Table 1:** Results from SelectScreen kinase profiling service (Invitrogen–Life Technologies)

**Compound**	**[ATP] tested (μM)**	**Kinase tested**	**IC_50_ (nM)**
*(A)*			
2-*O*-Bn-InsP_5_	100	PDK1	26.5
2-*O*-Bn-InsP_5_	75	Akt1 (PKB*α*)	>100000
2-*O*-Bn-InsP_5_	200	Akt2 (PKB*β*)	77000
2-*O*-Bn-InsP_5_	10	FRAP (mTOR)	1300
			
*(B)*			
InsP_5_	100	PDK1	613
InsP_5_	75	Akt1 (PKB*α*)	>100000
InsP_5_	200	Akt2 (PKB*β*)	38300
			
**Compound**	**[ATP] tested (μM)**	**Kinase tested**	**% Inhibition-mean**
*(C)*			
Ins(1,4,5)P_3_	100	PDK1	17
Ins(1,3,4,5)P_4_	100	PDK1	56
Ins(1,4,5,6)P_4_	100	PDK1	33
Ins(3,4,5,6)P_4_	100	PDK1	35
InsP_5_	100	PDK1	71
Ins(1,2,3,4,5,6)P_6_	100	PDK1	18

Abbreviations: InsP_5_=inositol 1,3,4,5,6-pentakisphosphate; 2-*O*-Bn-InsP_5_=2-*O*-benzyl-*myo*-inositol 1,3,4,5,6-pentakisphosphate.

10-point titration for 2-*O*-Bn-InsP_5_.

10-point titration for InsP_5_.

Single point for the indicated inositol polyphosphates. A concentration of 1 μM of the compounds was used in the assays.
