# Supplementary material for: A novel inhibitor of the PI3K/Akt pathway based on the structure of inositol 1,3,4,5,6-pentakisphosphate
Source: Br J Cancer. 2010 Jan 5;102(1):104–14. doi: 10.1038/sj.bjc.6605408 (PMC2813745; doi:10.1038/sj.bjc.6605408)
Supplement: Supplementary Table 1 [file 6605408x1.doc]

**Results from SelectScreen^TM^ Kinase Profiling Service**

**(Invitrogen-Life Technologies): Single Point for 2-*O*-Bn-InsP_5_**

|  | **[ATP] Tested (μM)** | **Kinase Tested** | **% Inhibition Mean** |
| --- | --- | --- | --- |
| 1 | 10 | ABL1 | 0 |
| 2 | 200 | AKT2 (PKBβ) | 16 |
| 3 | 100 | AKT3 (PKBγ) | 10 |
| 4 | 50 | AMPK A1/B1/G1 | 16 |
| 5 | 150 | AMPK A2/B1/G1 | 25 |
| 6 | 10 | AURKA (Aurora A) | 34 |
| 7 | 100 | BRAF | 5 |
| 8 | 25 | BTK | 5 |
| 9 | 10 | CAMK2A (CaMKII alpha) | -2 |
| 10 | 5 | CDC42 BPA (MRCKA) | 17 |
| 11 | 25 | CDK1/cyclin B | 9 |
| 12 | 25 | CDK2/cyclin A | 2 |
| 13 | 10 | CDK5/p25 | 3 |
| 14 | 10 | CDK5/p35 | 2 |
| 15 | 50 | CHEK1 (CHK1) | 15 |
| 16 | 75 | CHEK2 (CHK2) | 12 |
| 17 | 25 | CLK1 | -4 |
| 18 | 5 | CSNK1A1 (CK1α1) | 0 |
| 19 | 5 | CSNK1G1 (CK1γ1) | 7 |
| 20 | 50 | CSNK2A2 (CK2α2) | 2 |
| **21** | **10** | **FRAP1 (mTOR)** | **48** |
| 22 | 5 | GRK5 | 34 |
| 23 | 10 | GSK3A (GSK3α) | 3 |
| 24 | 10 | GSK3B (GSK3β) | 11 |
| 25 | 5 | IKBKB (IKKβ) | 13 |
| 26 | 25 | IRAK4 | -35 |
| 27 | 5 | ITK | -11 |
| 28 | 75 | JAK1 | -1 |
| 29 | 50 | LCK | 7 |
| 30 | 100 | MAP2K1 (MEK1) | 14 |
| 31 | 100 | MAP2K2 (MEK2) | 8 |
| 32 | 100 | MAPK1 (ERK2) | 6 |
| 33 | 100 | MAPK14 (p38α) | 30 |
| 34 | 50 | MAPK3 (ERK1) | 19 |
| 35 | 100 | MAPK8 (JNK1) | 24 |
| 36 | 100 | MAPK9 (JNK2) | -1 |
| 37 | 5 | MAPKAPK2 | 20 |
| 38 | 5 | MARK1 (MARK) | 0 |
| 39 | 10 | PAK6 | 9 |
| **40** | **100** | **PDK1** | **79** |
| 41 | 5 | PRKACA (PKA) | 4 |
|  | **[ATP] Tested (μM)** | **Kinase Tested** | **% Inhibition Mean** |
| 42 | 25 | PRKCA (PKCα) | 21 |
| 43 | 200 | PRKCB1 (PKCβI) | 11 |
| 44 | 25 | PRKCD (PKCδ) | 9 |
| 45 | 25 | PRKCE (PKCε) | 14 |
| 46 | 5 | PRKCZ (PKCζ) | 10 |
| 47 | 100 | RAF1 (cRAF) Y340D Y341D | 5 |
| 48 | 5 | ROCK1 | 7 |
| 49 | 50 | ROCK2 | 13 |
| 50 | 5 | RPS6KA1 (RSK1) | 2 |
| 51 | 10 | RPS6KB1 (p70S6K) | 17 |
| 52 | 50 | SRC | 4 |
| 53 | 150 | CDK7/cyclin H/MNAT1 | -2 |
| 54 | 25 | CDK9/cyclin T1 | -11 |
| 55 | 10 | CHUK (IKKα) | -2 |
| 56 | 5 | PI4KB (PI4Kβ) | 0 |
| 57 | 25 | PIK3CA/PIK3R1(p110α/p85α) | 2 |
| 58 | 25 | PIK3CD/PIK3R1(p110δ/p85α) | 6 |
| 59 | 25 | PIK3CG (p110γ) | -41 |

2-*O*-Bn-InsP_5_ was tested at a concentration of 1 μM.
