# Supplementary material for: A novel inhibitor of the PI3K/Akt pathway based on the structure of inositol 1,3,4,5,6-pentakisphosphate
Source: Br J Cancer. 2010 Jan 5;102(1):104–14. doi: 10.1038/sj.bjc.6605408 (PMC2813745; doi:10.1038/sj.bjc.6605408)
Supplement: Supplementary Table 2 [file 6605408x2.doc]

**Supplementary Table 2**

**Results from SelectScreen^TM^ Kinase Profiling Service**

**(Invitrogen-Life Technologies): Single Point for InsP_5_.**

|  | **[ATP] Tested (μM)** | **Kinase Tested** | **%**  **Inhibition Mean** |
| --- | --- | --- | --- |
| 1 | 50 | AMPK A1/B1/G1 | 4 |
| 2 | 150 | AMPK A2/B1/G1 | 7 |
| 3 | 10 | AURKA  (Aurora A) | 1 |
| 4 | 5 | GRK5 | 37 |
| 5 | 5 | IKBKB (IKKβ) | 1 |
| 6 | 100 | MAPK14 (p38α) | 23 |
| 7 | 5 | MAPKAPK2 | 4 |
| 8 | 100 | **PDK1** | **68** |

InsP_5_ was tested at a concentration of 1 μM.
